# HMGB1 regulates Th17 cell differentiation and function in patients with psoriasis

**DOI:** 10.1002/iid3.1205

**Published:** 2024-02-27

**Authors:** Xiaofeng Zhu, Yue Dou, Yawen Lin, Gaoping Chu, Jing Wang, Lei Ma

**Affiliations:** ^1^ Department of Dermatology Binzhou Medical University Hospital Binzhou China

**Keywords:** HMGB1, IL‐17A, IL‐23, psoriasis, Th17 cells

## Abstract

**Background:**

Psoriasis is an immune‐mediated chronic inflammatory skin disease, in which T helper 17 (Th17) cells and its effective cytokine interleukin (IL)‐17A play a pivotal pathogenic role. High mobility group box 1 (HMGB1) is an important proinflammatory cytokine, which has been confirmed to be highly expressed in the peripheral circulation and epidermis tissues of psoriasis patients. The regulatory effect of HMGB1 on IL‐17A expression and function has been reported in some inflammatory and autoimmune diseases by the HMGB1‐Toll‐like receptor 4 (TLR4)‐interleukin (IL)‐23‐IL‐17A pathway. While, in the pathological environment of psoriasis, whether HMGB1 can exert the regulatory effect on IL‐17A is not clear.

**Objective:**

We aimed to evaluate the role of HMGB1‐TLR4‐IL‐23‐IL‐17A pathway in the pathogenesis of psoriasis and explore the possible regulatory mechanism of HMGB1 on Th17 cell differentiation.

**Methods:**

Serum levels of HMGB1, TLR4, IL‐23, and IL‐17A were quantified in 50 patients with moderate‐to‐severe plaque psoriasis and 30 healthy controls. Peripheral blood mononuclear cells  were acquired from 10 severe psoriasis patients and administrated by different concentrations of recombinant‐HMGB1 (rHMGB1) to detect the Th17 cell percentage, mRNA and protein levels of TLR4, IL‐23, IL‐17A and retinoid‐related orphan receptor γt (RORγt).

**Results:**

The serum levels of HMGB1, TLR4, IL‐23, and IL‐17A in psoriasis patients were significantly higher than healthy controls, especially in severe patients, and positively correlated with the severity index. There were also positive correlations between every two detected indicators of HMGB1, TLR4, IL‐23, and IL‐17A. In vitro study, rHMGB1 can promote the elevated expression of Th17 cell percentage as well as TLR4, IL‐23, IL‐17A, and RORγt in a dose‐dependent manner.

**Conclusion:**

HMGB1 can contribute to the pathogenesis of psoriasis by regulating Th17 cell differentiation through HMGB1‐TLR4‐IL‐23‐RORγt pathway, then promotes IL‐17A production and aggravates inflammation process. Targeting HMGB1 may be a possible potential candidate for the immunotherapy of psoriasis.

## Graphical abstract

Our study demonstrates that HMGB1 can contribute to the pathogenesis of psoriasis by regulating Th17 cell differentiation through the HMGB1‐TLR4‐IL‐23‐RORγt pathway, then promote IL‐17A production and aggravate inflammation process. Targeting HMGB1 may be a possible potential candidate for the immunotherapy of psoriasis.

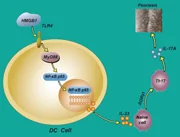

## 1 INTRODUCTION

Psoriasis is an immune‐mediated chronic and relapsing inflammatory skin disease, characterized by a dysregulation of the immune system with multifactorial pathogenesis. Immune cells infiltration, such as T cells, dendritic cells, macrophages, and neutrophils, is linked to the pathological characters of psoriasis.^1^ Therapeutic effect on mouse psoriatic inflammation by downregulating oxidative and inflammatory mediators in neutrophils and dendritic cells has been reported by Al‐Harbi et al.^2^ T helper 17 (Th17) cells and its effective cytokine interleukin (IL)‐17A play a pivotal pathogenic role in the development of psoriasis, and IL‐17A inhibitors also present excellent therapeutic effects for moderate‐to‐severe plaque psoriasis.^3^ IL‐23 is an upstream regulatory cytokine that acts early in the inflammatory cascade in psoriasis.^3^ The most important effect of IL‐23 in psoriasis lesions is the stabilization of a cytokine‐secreting pathogenic phenotype of Th17 cells^4^, ^5^ and the promotion of IL‐17 production by effector and memory T cells.^6^ Retinoid‐related orphan receptor γt (RORγt) is the specific transcription factor necessary for Th17‐cell lineage differentiation and function. It has been demonstrated that systemic IL‐23 signaling can upregulate the expression of RORγt in the context of psoriatic inflammation by Nadeem et al.^7^

High mobility group box‐1 (HMGB1), initially described as a nonhistone nuclear protein with transcriptional regulatory properties, is now recognized as a proinflammatory cytokine in the pathogenesis of various inflammatory and autoimmune diseases, including psoriasis.^8^, ^9^, ^10^, ^11^ HMGB1 has been confirmed to be highly expressed in psoriasis patients' serum and epidermis tissues.^9^, ^10^ Additionally, HMGB1 treatment can significantly aggravate psoriatic inflammation in imiquimod (IMQ)‐induced mouse psoriasis‐like inflammation,^12^ which further indicates that HMGB1 participates in the pathogenesis of psoriasis. Besides stimulating the immune system to produce an inflammatory response, HMGB1 is also involved in signal transduction, which can interact with Toll‐like receptors (TLRs) 2 and 4, as well as receptors for advanced glycation end products (RAGE) by activating the nuclear factor kappa B (NF‐κB) signal pathways, inducing the release of downstream inflammatory mediators.^13^, ^14^, ^15^ Th cells and other immune cells express TLRs and RAGE, so HMGB1 can act as an endogenous ligand for these receptors and regulate their function. Multiple studies have shown that HMGB1 can activate IL‐23 secretion by macrophages through binding to TLR4.^16^, ^17^, ^18^ In the murine asthma model study, HMGB1 has been confirmed to directly and indirectly induce Th17 immune response by TLR4‐NF‐κB signal pathway.^19^ And, in the study of renal ischemia–reperfusion injury, HMGB1 was demonstrated to activate macrophages to secrete IL‐23 by binding TLR4 and form the HMGB1‐TLR4‐IL‐23‐IL‐17A axis to further promote the inflammatory cascade reaction.^20^ However, whether HMGB1 can regulate Th17 cell differentiation within the pathological environment of psoriasis remains unclear. In the present study, we detected the serum levels of HMGB1‐TLR4‐IL‐23‐IL‐17A pathway in psoriasis patients, carried out in vitro experiments to explore the regulatory effects of HMGB1 on Th17 cell differentiation and IL‐17A expression, and tried to provide new ideas for the immune targeted therapy of psoriasis.

## 2 MATERIALS AND METHODS

### 2.1 Patients and controls

Fifty moderate‐to‐severe plaque psoriasis diagnosed by clinical features and/or histopathology (33 males and 17 females, aged 15–77 years old), psoriasis area and severity index (PASI) 4.4–37.8 were enrolled in the study. All the patients had not undergone systemic or local therapy within 1 month or 1 week, without pregnant, breastfeeding, psoriatic arthritis (previous/current signs and symptoms of joint involvement), or other concomitant systemic inflammatory diseases, such as lupus erythematosus and rheumatoid arthritis. Meanwhile, 30 age‐ and sex‐matched healthy volunteers (20 males and 10 females, aged 21–60 years old) were enrolled as healthy controls. This study was approved by the Ethics Committee of Binzhou Medical University Hospital (approval number: KYLL‐2022‐64), and written informed consents were obtained from all participants.

### 2.2 Blood samples collection and preparation

Serum samples were acquired from all psoriasis patients and healthy controls after an overnight fast in the morning between 8:00 and 9:00 a.m. Peripheral blood mononuclear cells (PBMCs) from 10 severity psoriasis patients were isolated and collected by Ficoll–Hypaque density gradient centrifugation, cultured at a cell concentration of 2 × 10^6^ cells/mL in RPMI 1640 medium (cat. no. E600028; Sangon Biotech108 Co., Ltd.) supplemented with 10% fetal bovine serum (cat. no. E510008‐0500; Sangon Biotech Co., Ltd.), and 1% penicillin–streptomycin liquid (cat. nos. P1400, Beijing Solarbio Science & Technology Co., Ltd.), then stimulated and cocultured with different concentrations of rHMGB1 (0, 10, 100, 200, and 400 ng/mL, cat. no. 557804; Biolegend), respectively at 37°C for 24 h in a humidified incubator with a 5% CO_2_ for subsequent flow cytometric analysis, real‐time quantitative reverse transcription‐polymerase chain reaction (RT‐PCR) and western blot detection. All the experiments were repeated three times.

### 2.3 Enzyme‐linked immunosorbent assay (ELISA) for HMGB1, TLR4, IL‐23, and IL‐17A

The serum concentrations of HMGB1, TLR4, IL‐23, and IL‐17A of psoriasis patients and healthy controls were measured by ELISA according to the manufacturer's instructions for three repeated experiments (cat. no E‐EL‐H1554C, E‐EL‐H5820C, E‐EL‐H0107C, and E‐El‐H5812C; Wuhan Elabscience Biotechnology).

### 2.4 Flow cytometric analysis for Th17 cell percentage

To analyze the Th17 cell percentage (CD4^+^IL17^+^ T cells/CD4^+^ T cells%), PBMCs were first stimulated with 50 ng/mL phorbol 12‐myristate 13‐acetate (PMA, cat. no. P6741; Beijing Solarbio Science & Technology Co., Ltd.), 1 μg/mL ionomycin (cat. no. 18800; Beijing Solarbio Science & Technology Co., Ltd.), and 10 μg/mL brefeldin A (cat. no; 7150022; Beijing Solarbio Science & Technology Co., Ltd.) at 37°C under a 5% CO_2_ environment for 4 h. Then, cells were stained with APC‐labeled anti‐CD4 antibody (0.8 μg/mL, cat. no. 357408; BioLegend Inc.) in the dark at room temperature for 30 min. After surface staining, the cells were fixed and permeabilized in a dark environment at room temperature and stained with PE‐labeled anti‐IL‐17A antibody for 30 min (2.5 μg/mL, cat. no. 512306; BioLegend Inc.). Flow cytometric analysis was performed on a LSRII flow cytometer (BD Biosciences) and analyzed by FlowJo software (FlowJo‐v 10.8.1.).

### 2.5 Real‐time quantitative RT‐PCR analysis for TLR4, RORγt, IL‐23 and IL‐17A mRNA expression levels

Total RNA was extracted from PBMCs using Trizol (cat. no. 133 B511311; Sangon Biotech Co., Ltd.) following the manufacturer's instructions. Complementary DNA was synthesized by Evo M‐MLV Mix Kit with gDNA Clean (cat. no. AG11728; Hunan Accurate Biotechnology Co., Ltd.). The mRNA expression levels of TLR4, RORγt, IL‐23, and IL‐17A were quantified by RT‐qPCR® Green Premium Pro Taq136 HS qPCR Kit (cat. no. AG11701; Hunan Accurate Biotechnology Co., Ltd.) on the CFX96137 Touch™ Real‐time PCR detection system (Bio‐Rad Laboratories Inc.). The primers were designed by China Hunan Precision Biotechnology Co., Ltd. and listed in Table 1. β‐actin was used as the endogenous reference gene, and the expression level of the interest gene was calculated using the following formula:

$$2^{-[\text{Ct}(\text{interest gene})-\text{Ct}(\text{endogenous reference gene})\;\text{of sample}]-[\text{Ct}(\text{interest gene})-[\text{Ct}(\text{endogenous reference gene})\;\text{of calibrate}]}.$$

**TABLE 1:** Primers used for quantitative real‐time PCR.

Gene	Primer sequence
TLR4	Sense, 5ʹ‐GAAGGGGTGCCTCCATTTCAG‐3ʹ and antisense, 5ʹ‐GCTGGGACACCACAACAATCA‐3ʹ
IL‐23	Sense, 5ʹ‐TGGGAGACTCAGCAGATTCCA‐3ʹ and antisense, 5ʹ‐ GGAGGCTGCGAAGGATTTTGA‐3ʹ
RORγT	Sense, 5ʹ‐AGGAGCAATGGAAGTGGTGCT‐3ʹ and antisense, 5ʹ‐ CTCCATGCCACCGTATTTGCC‐3ʹ
IL‐17A	Sense, 5ʹ‐CAACCGATCCACCTCACCTTG‐3ʹ and antisense, 5ʹ‐ TGTGGTAGTCCACGTTCCCAT‐3ʹ
ACTIN	Sense, 5ʹ‐ TGGCACCCAGCACAATGAA‐3ʹ and antisense, 5ʹ‐ CTAAGTCATAGTCCGCCTAGAAGCA‐3ʹ

Abbreviation: PCR, polymerase chain reaction.

### 2.6 Western blot analysis for TLR4, RORγt, IL‐23, and IL‐17A protein levels

Total protein was extracted using RIPA lysis buffer (cat. no. R0020; Beijing Solarbio Science & Technology Co., Ltd.). Equal quantities of denatured protein (30 μg) were separated by 10% or 12.5% sodium dodecyl sulfate‐polyacrylamide gel electrophoresis and then transferred to polyvinylidene fluoride membranes (cat. no. ipvh00010; Sigma‐Aldrich, Merck KGaA). After blocking in 5% skim milk for 60 min, the membranes were incubated with primary antibodies against RORγt, IL‐23, and IL‐17A (all 1:1000 dilution, cat. no. ab113434, ab45420, and ab13556, Abcam) and TLR4 antibody (1:500 dilution, cat. no. sc‐293072; Santa Cruz) at 4°C overnight and then incubated with the corresponding peroxidase‐conjugated goat anti‐rabbit IgG antibodies (1:10,000 dilution, cat. no. ZB‐2306, ZSGB‐BIO) for 1 h at room temperature. β‐actin antibody (1:10,000 dilution, cat. no. ab179467, Abcam) was used to confirm equal protein loading in each lane. The protein bands were detected by an ECL kit (cat. no. MA0186, Dalian Meilun Biology Technology Co., Ltd.) and analyzed with ImageJ software v 4.1 (National Institutes of Health).

### 2.7 Statistical analysis

Shapiro–Wilk test was employed to detect the normality of the data, which were expressed as mean ± standard deviation for normally distributed data or median [25th–75th percentile] for non‐normally distributed data. The differences between psoriasis patients and healthy controls were compared by independent‐sample *t*‐test and Mann–Whitney *U* test, respectively. Pearson correlation and Spearman's test were used to conduct correlation analysis. According to Levene's test of homogeneity of variance, one‐way analysis of variance was used to compare the differences among in vitro experimental data for homogeneous variance, followed by the least‐significant difference LSD test performing multiple comparisons. Statistical analysis was conducted using the SPSS 27.0 (IBM Corp.) and GraphPad Prism 5 (GraphPad Software Inc.). The *p*‐value of <.05 was considered statistically significant.

## 3 RESULTS

### 3.1 Serum concentrations of HMGB1, TLR4, IL‐23, and IL‐17A

**FIGURE 1:**
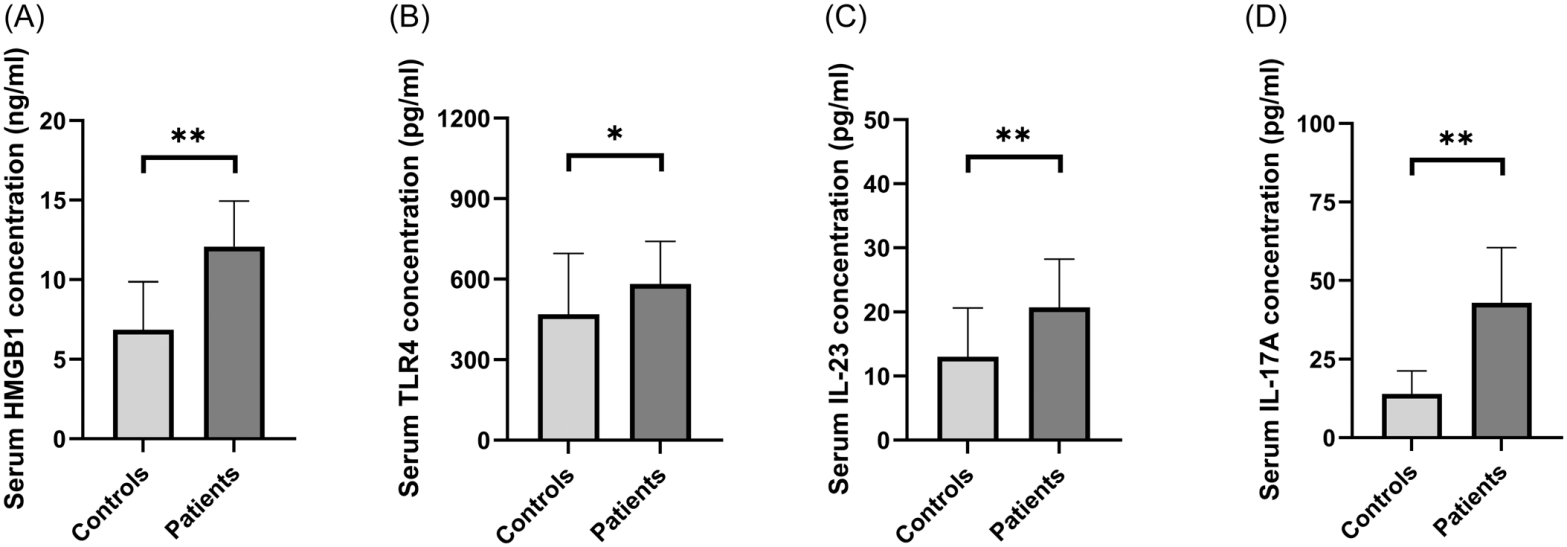
Comparison of the serum levels of HMGB1, TLR4, IL‐23, and IL‐17A between psoriasis patients (*n* = 50) and healthy controls (*n* = 30). HMGB1 (A), TLR4 (B), IL‐23 (C), and IL‐17A (D) serum levels of psoriasis patients were significantly higher than those of healthy controls. *p < .05 and **p < .01.

**FIGURE 2:**
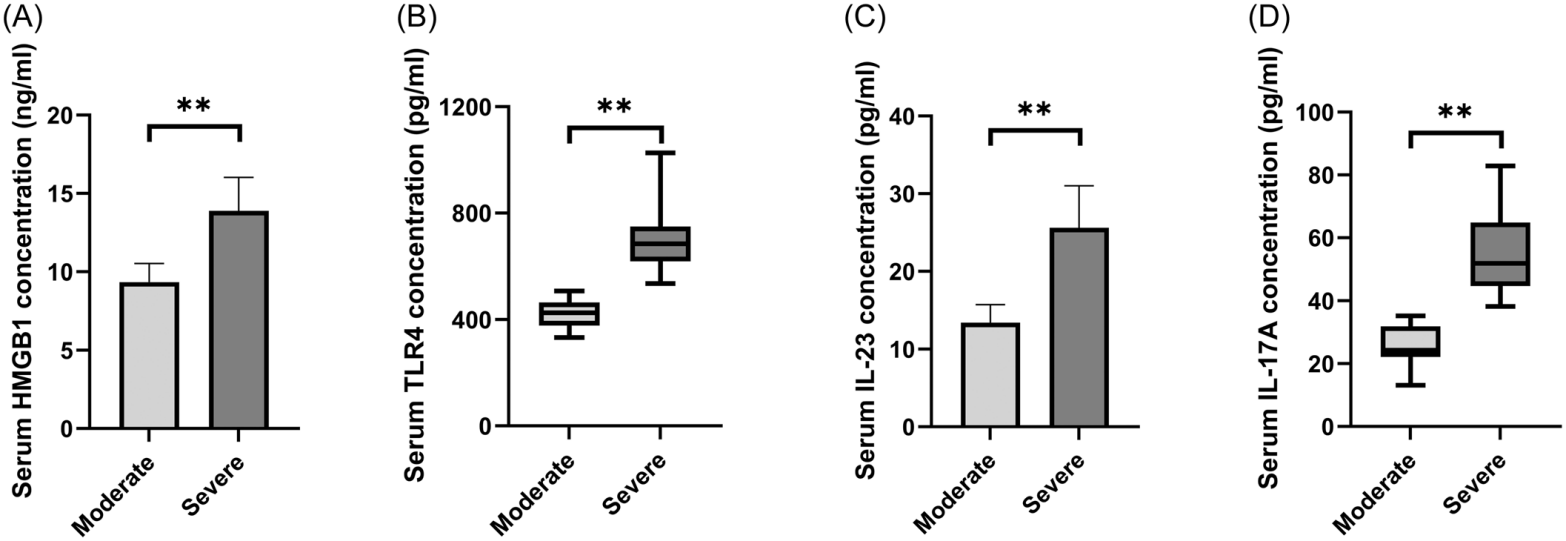
Comparison of the serum levels of HMGB1, TLR4, IL‐23, and IL‐17A between moderate (*n* = 20) and severe (*n* = 30) psoriasis patients. HMGB1 (A), TLR4 (B), IL‐23 (C), and IL‐17A (D) serum levels of severe patients were significantly higher than those of moderate patients. **p < .01.

HMGB1, TLR4, IL‐23, and IL‐17A serum concentrations in psoriasis patients were significantly elevated compared with those in healthy controls (*t* = 7.716, 2.433, 4.443, and 10.339, respectively, all *p* < .05 or .01, Table 2, Figure 1A–D). Further comparisons between psoriasis patients showed that the serum concentrations of HMGB1, TLR4, IL‐23, and IL‐17A in severe psoriasis patients were all obviously higher than those of moderate psoriasis patients (Table 2 and Figure 2A–D), HMGB1 (*t* = 9.651, *p* < .001), TLR4 (*z* = −5.94, *p* < .001), IL‐23 (*t* = 10.94, *p* < .001), and IL‐17A (*z* = −3.69, *p* < .001).

**TABLE 2:** Expression levels of serum HMGB1, TLR4, IL‐23, and IL‐17A in patients with psoriasis vulgaris and healthy control, which are denoted by ($\bar{x}$ ± s) or M (P25, P75).

Groups	*n*	PASI scores	HMGB1	TLR4	IL‐23	IL‐17A
Healthy control	30	–	6.86 ± 3.03	469.9 ± 225.73	13.0 ± 7.62	13.93 ± 7.37
Psoriasis patients	50	13 (6.1,16.65)^a^	12.08 ± 2.88^a^	583.76 ± 156.78^a^	20.74 ± 7.48	42.99 ± 17.45^a^
Moderate patients	20	5.76 ± 1.42^a^	9.36 ± 1.67^a^	425.63 (377.83,464.73)^a^	13.41 ± 2.34	24.33 (22.15,31.88)^a^
Severe patients	30	16.3 (13.2,17.25)^b^	13.89 ± 2.14^b^	684.04 (618.79,749.3)^b^	25.62 ± 5.4	51.87 (44.75,64.9)^b^

*Note*: Compared with healthy controls, ^a^ *p* < .05, compared with moderate patients, ^b^ *p* < .05.

Abbreviation: PASI, psoriasis area and severity index.

### 3.2 Correlation analysis in psoriasis patients

**FIGURE 3:**
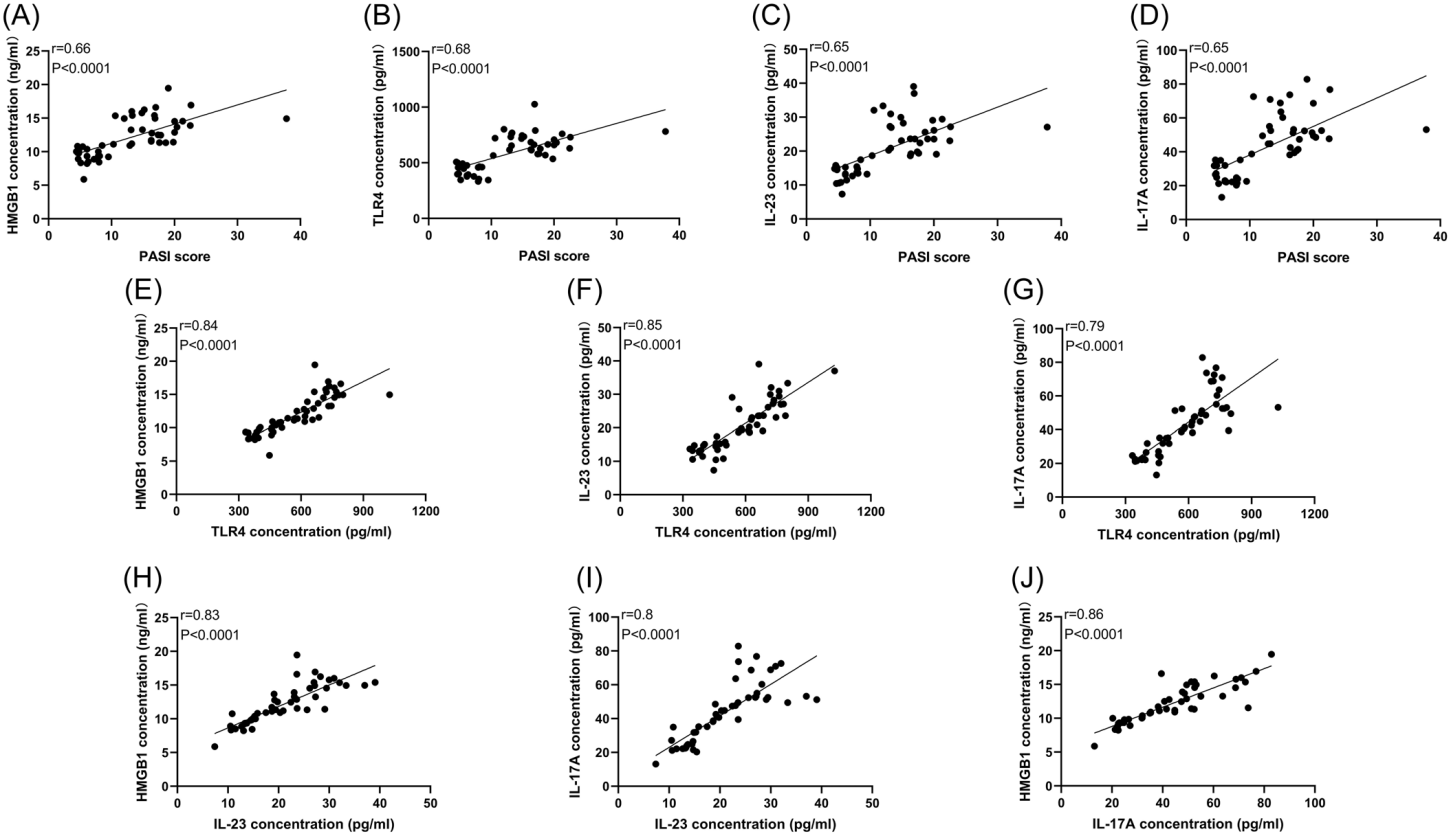
Correlation analysis of PASI, HMGB1, TLR4, IL‐23, and IL‐17A in psoriasis patients. (A–D) PASI correlation analysis with HMGB1, TLR4, IL‐23, and IL‐17A. (E–G) HMGB1 correlation analysis with TLR4, IL‐23, and IL‐17A. (H, I) TLR4 correlation analysis with IL‐23 and IL‐17A. (J) IL‐23 correlation analysis with IL‐17A. All *p* < .001, *n* = 50.

Serum concentration of HMGB1, TLR4, IL‐23, and IL‐17A were all positively correlated with psoriasis patients' PASI (*r* = 0.66, 0.68, 0.65, and 0.65, respectively, all *p* < .001, Table 3, Figure 3A–D). In addition, there was also a positive correlation between every two detected indexes among HMGB1, TLR4, IL‐23, and IL‐17A in psoriasis patients (Table 3, Figure 3E–J).

**TABLE 3:** Correlation analysis of PASI, HMGB1, TLR4, IL‐23, and IL‐17A in psoriasis patients.

Items	HMGB1	TLR4	IL‐23	IL‐17A
PASI scores	0.66^a^	0.68^a^	0.65^a^	0.65^a^
TLR4	0.84^b^	–	0.85^b^	0.79^b^
IL‐23	0.83^c^	–	–	0.8^c^
IL‐17A	0.86^d^	–	–	–

*Note*: Correlation with PASI scores ^a^ *p* < .001, correlation with TLR4 ^b^ *p* < .001, correlation with IL‐23 ^c^ *p* < .001, correlation with IL‐17A ^d^ *p* < 0.001.

Abbreviation: PASI, psoriasis area and severity index.

### 3.3 rHMGB1 promotes Th17 cell percentage and its effective cytokine IL‐17A production in psoriasis patients' PBMCs

**FIGURE 4:**
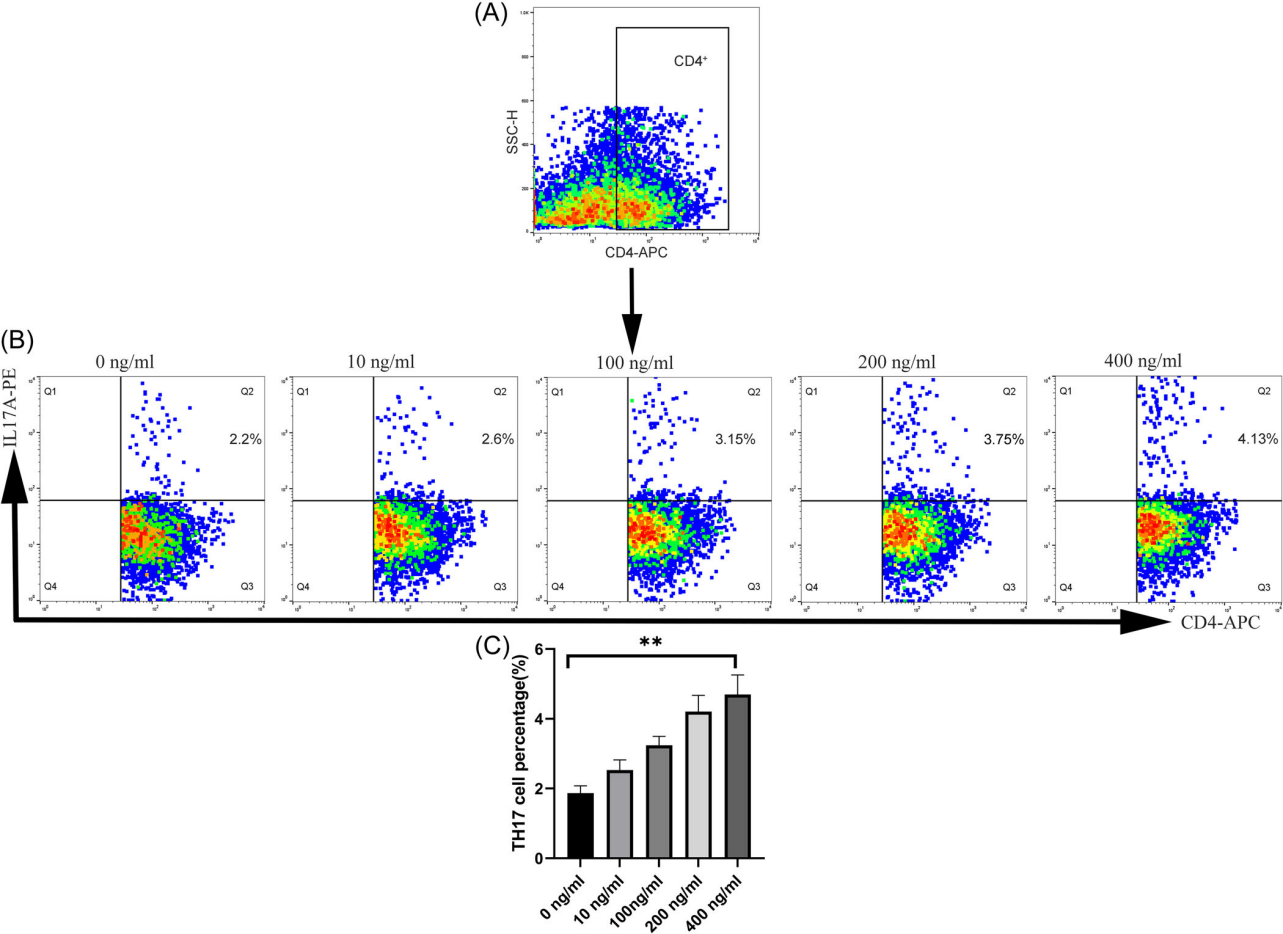
Flow cytometric analysis of Th17 cell percentage in psoriasis patients' peripheral blood mononuclear cells by rHMGB1 stimulation (*n* = 10). (A) The CD4^+^ population of lymphocytes was selected by APC‐labeled anti‐CD4 antibody from peripheral blood mononuclear cells. (B) The respective flow cytometry results of Th17 cell percentage after treatment by different concentrations of rHMGB1 (0, 10, 100, 200, and 400 ng/mL) in vitro. (C) The quantitative analysis results were listed and compared. All the experiments were repeated three times. **p < .01.

**FIGURE 5:**
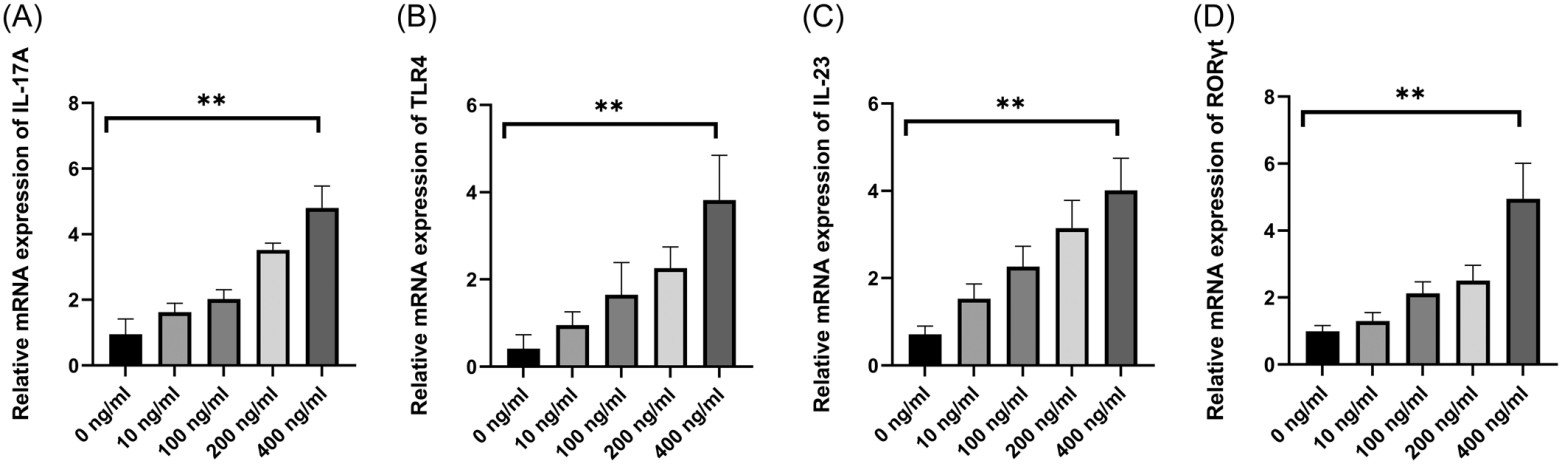
rHMGB1 enhanced the messenger RNA (mRNA) expression of IL‐17A, TLR4, IL‐23, and RORγt in psoriasis patients' peripheral blood mononuclear cells (*n* = 10). mRNA expression levels of IL‐17A (A), TLR4 (B), IL‐23 (C), and RORγt (D) after treatment by different concentrations of rHMGB1 (0, 10, 100, 200, and 400 ng/mL) in vitro. All the experiments were repeated three times. **p < .01.

**FIGURE 6:**
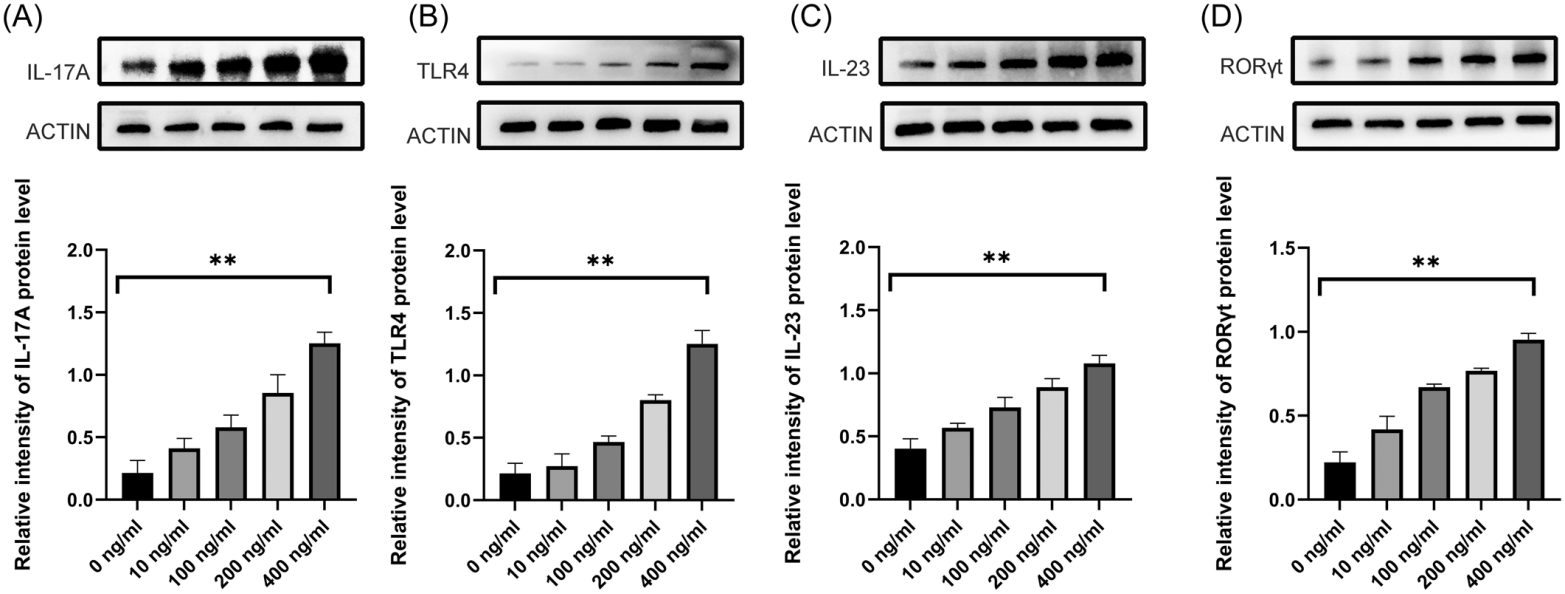
rHMGB1 elevated the protein expression levels of IL‐17A, TLR4, IL‐23, and RORγt in psoriasis patients' peripheral blood mononuclear cells (*n* = 10). Protein expression levels of IL‐17A (A), TLR4 (B), IL‐23 (C), and RORγt (D) after treatment by different concentrations of rHMGB1 (0, 10, 100, 200, and 400 ng/mL) in vitro. Representative western blot bands were listed in the top location, and the quantitative analysis results were listed in the bottom location. All the experiments were repeated three times. **p < .01.

Th17 cell percentage was presented as IL‐17A^+^CD4^+^ cells gating on CD4^+^ cells. After rHMGB1 treatment for 24 h in vitro, Th17 cell percentages of psoriasis patients' PBMCs were significantly increased in a dose‐related fashion with rHMGB1 concentration (0, 10, 100, 200, and 400 ng/mL, *F* = 55.39, all *p* < .001, Figure 4). Consistent with the increased Th17 cell percentage, rHMGB1 treatment resulted in a dose‐dependent promotional effect on IL‐17A mRNA and protein expression levels in psoriasis patients' PBMCs (*F* = 83.94 and *F* = 36.13, both *p* < .001, Figures 5A and 6A).

### 3.4 rHMGB1 upregulates the expression levels of TLR4 and IL‐23, in psoriasis patients' PBMCs

As shown in Figures 5B,C and 6B,C, both the mRNA and protein expression levels of TLR4 and IL‐23 were upgraded in a dose‐related manner following the increased rHMGB1 stimulating concentration (TLR4: *F* = 25.58 and *F* = 39.58, IL‐23: *F* = 87.08, *F* = 46.61, all *p* < .001).

### 3.5 rHMGB1 promotes the expression levels of Th17 cell‐specific transcription factor RORγt

To further determine whether the increased Th17 cell percentage and IL‐17A expression levels in the presence of rHMGB1 were associated with Th17 cell‐specific transcription factor RORγt, the mRNA and protein expression levels were measured. In accordance with the tendency of Th17 cell percentage and IL‐17A levels, rHMGB1 treatment can significantly promote RORγt mRNA and protein expression levels (*F* = 43.43 and *F* = 103.37, both *p* < .001, Figures 5D and 6D).

## 4 DISCUSSION

Psoriasis is a chronic immune‐mediated inflammatory disease, in which IL‐17A is regarded as a preponderant inflammatory cytokine mediating its pathogenesis. HMGB1, an important proinflammatory cytokine, has been reported to exert a regulatory effect on Th17 immune response,^19^ but this effect has not been confirmed in the psoriatic pathological conditions. Therefore, the present study aimed to further evaluate the pathogenic role of HMGB1 in psoriasis by exploring the possible regulatory effect of HMGB1 on Th17 cell differentiation, which revealed that HMGB1 can promote Th17 cell differentiation and IL‐17A production in a dose‐dependent manner in the PBMCs from psoriasis patients and supplemented data for HMGB1‐based therapeutic strategies.

In this study, we first confirmed that HMGB1 was significantly increased in the peripheral circulation of psoriasis patients, which is consistent with previous reports.^9^, ^10^ In addition, HMGB1 serum levels were obviously higher in severe patients than those in moderate patients and, more importantly, positively correlated with the index of psoriasis severity degree, which further demonstrates that HMGB1 is involved in the development and progress of psoriasis. The crucial role of IL‐17A in the pathological conditions of psoriasis has been strongly supported,^3^, ^21^, ^22^ which is produced predominantly by Th17 cells.^23^, ^24^ Our previous studies have confirmed the increased percentage of Th17 cells and expression levels of IL‐17A in the peripheral blood of psoriasis patients.^25^ HMGB1 promoting IL‐17A release has been shown in myocardial ischemia–reperfusion injury.^26^ In this present study, significantly increased serum IL‐17A levels were demonstrated again and positively correlated with HMGB1 expression levels, which indicates that HMGB1 may exert a regulatory effect on IL‐17A expression.

IMQ‐induced psoriasis‐like inflammation is a classic animal model of psoriasis, which is mediated via the IL‐23/IL‐17 axis.^27^ Similar to the abundant cytoplasmic expression of HMGB1 in the lesional skin of psoriasis patients, IMQ‐induced psoriatic mice presented high HMGB1 cytoplasmic levels.^12^ Wild‐type mice intradermally injection with rHMGB1 resulted in epidermal thickening; moreover, administration of HMGB1 into IMQ‐induced lesional skin can further worsen the severity of psoriasis‐like inflammation.^12^ In addition, HMGB1 can augment the infiltration of immunocytes in lesions, such as T cells, neutrophils and dendritic cells, and trigger inflammatory mediators, including IL‐23/IL‐17A axis and the transcription factor RORγt.^12^, ^28^ TLR4, as a pattern recognition receptor, plays a crucial role in the induction of the inflammatory response and links to the activation of downstream signaling in several cell types.^29^, ^30^, ^31^ IL‐23 is the pro‐inflammatory cytokine and essential for the differentiation of Th17 lymphocytes.^32^ HMGB1 blockade and TLR‐4 deficiency can both significantly reduce the production of IL‐23 and IL‐17A; furthermore, exogenous IL‐23 administration can abrogate the decreased IL‐17 expression induced by HMGB1 blockade, which indicates that HMGB1‐TLR‐4 pathway contributes to IL‐17A secretion dependent on IL‐23 and forms HMGB1‐TLR4‐IL‐23‐IL‐17A pathway.^26^ However, in pathological conditions of psoriasis, it is still unclear whether the HMGB1‐TLR4‐IL‐23‐IL‐17A pathway may be involved in and contribute to the IL‐17A aggregation and its inflammatory reaction.

In this study, we detected the increased serum levels of HMGB1, TLR4, IL‐23, and IL‐17A and evaluated the positive correlation between HMGB1 and TLR4, HMGB1 and IL‐23, HMGB1 and IL‐17A, as well as TLR4 and IL‐23 and TLR4 and IL‐17A, which indicates that HMGB1‐TLR4‐IL‐23‐IL‐17A pathway may produce a pathogenic effect on psoriasis. To further explore the role of HMGB1 in the pathogenesis of psoriasis, different concentrations of rHMGB1 were exerted on PBMCs from psoriasis patients in vitro. HMGB1 performs its functions via interactions with TLRs.^33^, ^34^ The expression levels of TLR4 were found to be obviously increased when a higher concentration of rHMGB1 was administrated, which is similar to in vitro study of asthma.^22^ Previous studies have shown that rHMGB1 can stimulate splenic CD4^+^ T cells, augment the ratio of CD4^+^ IL‐17^+^ T cells, and lead to a significant increase of Th17 cell transcription factor RORγt and effective cytokine IL‐17 expression levels.^35^, ^36^, ^37^ And, in the study of asthma, HMGB1 has been demonstrated to induce Th17 cell differentiation by directly acting on naïve T cells to induce polarization and indirectly mediating the maturation and antigen‐presenting ability of dendritic cells to promote Th17 cell differentiation.^19^ RORγt is required for the effective induction, development, and function of Th17 cells.^38^, ^39^ Our present study showed that the Th17 cell percentage and RORγt expression levels were gradually elevated following the increased rHMGB1 administration concentration, which indicates that rHMGB1 can promote Th17 cell differentiation in the pathological environment of psoriasis. IL‐23, produced mainly by activated macrophages in response to TLR activation, plays an important bridge role in inducing Th17 cell differentiation and function.^40^, ^41^, ^42^ Studies on renal ischemia–reperfusion injury have shown that HMGB1 can stimulate the activation of macrophages and provoke the generation of IL‐23 in a TLR‐4‐dependent manner.^20^ In the present study, we confirmed that both HMGB1 and TLR4 serum levels were positively correlated with IL‐23 in psoriasis and rHMGB1 can dose‐dependently increase its expression levels, further indicating the regulatory effect of HMGB1 on Th17 cell differentiation. In addition, Th17 cell differentiation is medicated by multiple factors, among which IL‐1β and IL‐6 are essential for human Th17 cell differentiation.^43^ HMGB1 has been reported to contribute to the secretion of IL‐6 and IL‐1β by macrophages and sustain the inflammatory reaction,^44^ which may be another approach way for HMGB1 regulating Th17 cell differentiation. Previous research about the regulatory effect exerted on macrophages from dental pulp tissue specimens of pulpitis and healthy pulps by HMGB1 stimulation has shown that compared with HMGB1‐unstimulated cells, the IL‐17 levels in culture supernatants were significantly increased in the case of HMGB1 stimulation, especially in pulpitis samples,^45^ which further indicates that the promotional effect of HMGB1 on Th17 cell differentiation is an important immune regulatory pathway in pathological conditions. On the contrary, IL‐17A and HMGB1 have been shown to regulate mutually to exhibit a synergistic effect. The study on oxygen–glucose deprivation reported that the downregulation of HMGB1 expression caused a reduced IL‐17A expression, while the downregulation of IL‐17A expression resulted in a reduction of HMGB1 expression.^46^ Our previous study also confirmed that the increased serum levels of HMGB1 were significantly decreased after IL‐17A inhibitor Secukinumab induction treatment. So, there may exist a feedback loop between HMGB1 and IL‐17A, which can promote reciprocally and enhance the inflammatory effects synergistically.

Psoriatic inflammation is associated with numerous comorbidities, in which pathological parameters of psoriasis may mediate the potential mechanisms, including endoplasmic reticulum stress, pro‐inflammatory cytokine releases, excess production of reactive oxygen species, alterations in adipocytokine levels, and gut microbiota dysbiosis.^47^ Al‐Harbi et al. have reported that psoriasis can induce renal dysfunction by upregulation of NADPH oxidases and inducible nitric oxide synthase and mediate hepatic inflammation by oxidative stress and pro‐inflammatory cytokines production with IL‐17RC/NF‐κB signaling.^48^, ^49^ So, as a systemic disease, the mechanism of psoriasis is complex, interactive, and integrated, including innate and adaptive immune systems, and multiple and interweaving pathogenic factors contribute to its development and aggravation. Inspiringly, Bruton's tyrosine kinase inhibitor was reported to suppress IMQ‐induced psoriasis‐like inflammation through regulation of IL‐23/IL‐17A in innate immune cells,^50^ which enriches the recognition of psoriasis's pathogenesis and treatment idea.

In summary, there are several limitations in this study. First, we did not design to detect the regulatory effect of HMGB1 on PBMCs of healthy controls. Second, although HMGB1 has the potential to promote Th17 cell differentiation and induce IL‐17 expression in PBMCs of psoriasis patients by IL‐23 and TLR4 signaling pathways, more detailed mechanisms need to be explored and explained. Third, intervention experiments by drug blockade and genetic modification are required to further confirm the possible therapeutic treatment of HMGB1 both in human samples and animal models.

However, our present study provides further evidence that HMGB1 participates in the pathogenesis of psoriasis, which may exert its regulatory effect on Th17 cell differentiation and IL‐17A production by IL‐23 and TLR4 signaling pathways to amplify its inflammatory effects. So, inhibiting HMGB1 may be a candidate idea for the treatment of psoriasis. Indeed, to further identify the underlying mechanisms, more detailed and specific intervention experiments need to be established.

## AUTHOR CONTRIBUTIONS

Lei Ma was responsible for the design of this study and reviewed and edited this article. Xiaofeng Zhu carried out the experiments, performed the statistical analysis, and wrote the first draft of the manuscript. YD, Yue Dou, Yawen Lin, and Gaoping Chu performed the experiments. JW Jing Wang collected the clinical data. All authors read and approved the final manuscript, and agreed to be accountable for all aspects of the work in ensuring that questions related to the accuracy or integrity of the work are appropriately investigated and resolved.

## CONFLICT OF INTEREST STATEMENT

The authors declare no conflicts of interest.

## ETHICS STATEMENT

The study was reviewed and approved (approval no. KYLL‐2022‐64) by the Ethics Committee of Binzhou Medical University Hospital (Binzhou, China).

## Data Availability

The data that support the findings of this study are available on request from the corresponding author upon reasonable request.
